# Regional Heterogeneity in Vastus Lateralis Architecture Influences Fascicle Behavior During In Vivo Contractions

**DOI:** 10.1111/sms.70103

**Published:** 2025-07-09

**Authors:** Michele Trinchi, Baptiste Bizet, Paola Zamparo, Andrea Monte

**Affiliations:** ^1^ Department of Neurosciences, Biomedicine and Movement Sciences University of Verona Verona Italy

**Keywords:** belly gearing, F–V relationship, muscle behavior, muscle mechanics, regional differences

## Abstract

Skeletal muscle is heterogeneous in its architecture, with regional differences in fiber length and pennation angle that make up anatomically distinct regions. This study aimed to understand how these regional differences influence vastus lateralis (VL) behavior during isokinetic contractions in vivo. Knee extensor torque was measured in twelve healthy young adults using an isokinetic dynamometer during maximal contractions at different angular velocities (30° s^−1^, 75° s^−1^, 150° s^−1^, 210° s^−1^, 270° s^−1^). The fascicle length of VL was recorded by two ultrasound devices in its distal and middle regions, and muscle‐belly length was calculated as the longitudinal length change in the muscle belly. Fascicle (Vf) and muscle‐belly (Vm) velocities were calculated as the first derivative of the length–time curve in the phase at constant angular velocity. Muscle‐belly gearing (Gb) was calculated as Vm/Vf. At rest, greater thickness and pennation angles and lower fascicle lengths were observed in the middle vs. distal regions. During contraction, Vf and Vm increased as a function of angular velocity in both the investigated regions. The distal regions showed higher Vf and Vm values at all the investigated angular velocities. Significant differences in Gb were observed between regions but not as a function of knee angular velocity. Our data indicate that the architectural differences within a muscle affect the behavior of the active components during contraction. These results could help develop new musculoskeletal models to predict the muscle's mechanical output better.

## 1 Introduction

The shortening velocity of the muscle fascicles strongly affects a muscle's mechanical output and, consequently, the energetics of contraction. Indeed, the force–velocity (F–V) relationship is considered (along with the force–length relationship) an essential constraint on muscle performance [1, 2]. For this reason, it remains one of the most fundamental and best‐studied properties of skeletal muscle.

In humans, several factors could affect the parameters of the F–V relationships (e.g., maximum shortening velocity, maximum force, and curvature); muscle architecture plays a relevant role. For example, fascicle length could affect maximum shortening velocity (i.e., the longer the fascicle, the higher the number of sarcomere in‐series and, thus, the maximum shortening velocity [3]), while changes in pennation angle could potentially influence the muscles' force capacity (i.e., the higher the pennation angle, the higher the in‐parallel active components and, thus, the maximum isometric force) [4]. However, understanding the relationship between the mechanical output of a given muscle and its architecture could be complicated by the presence of regional heterogeneity since fascicle length and orientation can differ from one region to another [5].

Blazevich et al. [6] demonstrated that muscle architecture varies significantly along a muscle length (in vastus lateralis, medialis, intermedius, and rectus femoris), with shorter fascicles and higher pennation angles in the proximal/middle region compared to the distal; this has implications for the accuracy of models that assume a uniform intramuscular architecture. In addition, O'Brien et al. [7] indicated that intramuscular architecture differences also depend on the maturation stage of a subject.

These regional differences can be expected to influence mechanical output during contraction (e.g., fiber's contraction velocity and hence the F–V relationship), which was indeed observed in animal preparations. For example, Tijs et al. [8] observed significant differences between distal and proximal fascicle shortening velocities in rat medial gastrocnemius muscle during maximal and submaximal evoked contraction. Azizi and Deslauries [9] showed similar results in turkey and used a simple geometrical model to estimate the regional fascicle behavior of a “generic” pennate muscle; they observed that variations in pennation angle along a muscle could affect fascicle length changes, with higher expected modifications at lower angles of pennation, and concluded that regional variations in muscle and fascicle behavior can arise from the muscle's architectural features. These intraregional differences could also relate to differences in belly gearing. Belly gearing is a pennate muscle's capability to uncouple the muscle belly's behavior from that of its fascicles [10]. Indeed, it was observed that during low‐force and high‐velocity contractions, the muscle shortening velocity is higher compared to the fascicle shortening velocity, allowing the muscle to circumvent the limits imposed by the fascicle properties [11, 12, 13]. Since belly gearing is affected by resting pennation angle, it can be expected to differ between the proximal/middle and distal regions of a muscle, with several potential mechanical and physiological implications [5, 12, 14].

To our knowledge, the effects of regional architectural differences during in vivo contractions have not yet been investigated in humans, so this was the primary aim of this study. We focused on a pennate muscle (the vastus lateralis, VL) to examine whether regional differences imply differences in this muscle's capacity to uncouple its behavior from that of its fascicles.

Hence, in this study, we combined dynamometric and in vivo ultrasound evaluations to investigate the operating shortening velocity of the middle and distal fascicles of the vastus lateralis and the capacity of the muscle‐belly to uncouple its behavior from that of its fascicles in different regions.

Since the fascicle length is shorter in the middle than in the distal region [5, 6], we expected a higher shortening velocity for the muscle fascicle in the distal region.

Since belly gearing is affected by resting pennation angles [10, 15] and since the pennation angle is higher in the middle compared to the distal region, we also expected a higher belly gearing in the VL middle region compared to the distal one.

## 2 Methods

### 2.1 Participants

Twelve healthy men (age: 24 ± 1 years; body mass: 71.5 ± 7.4 kg; stature: 1.77 ± 0.7 m) participated in this study. All participants were moderately active (involved in recreational sports activities such as running or cycling) and trained 2–3 times per week. Subjects with neuromuscular injuries within the last six months were excluded from the study. All participants received written and oral instructions before the study and gave their written informed consent to the experimental procedure. The experimental protocol was approved by the Ethical Committee of the University of Verona (protocol number: 2019‐UNVRCLE‐0193291).

### 2.2 Experimental Design

Each subject participated in one experimental session. After five minutes of warm‐up and familiarization, the middle and distal VL muscle geometry was evaluated at rest and during isokinetic contractions using two ultrasound apparatuses synchronized with an isokinetic dynamometer.

### 2.3 Data Collection

The participants were secured on a dynamometer (Cybex HUMAC Norm, USA) using a pelvic and trunk Velcro strap, with their arms crossed in front of their chest. The dynamometer and the knee axis of rotation were aligned at 90° of knee flexion during a maximal voluntary contraction (MVC), as Bakenecker et al. [16] proposed to ensure alignment during contraction.

After a warm‐up based on submaximal fixed‐end and iso‐velocity contractions, resting muscle geometry was analyzed while the subjects were seated on the dynamometer with the knee angle set at 75°. After that, each participant performed three maximum isokinetic contractions at five different angular velocities (30° s^−1^, 75° s^−1^, 150° s^−1^, 210° s^−1^, and 270° s^−1^). During these contractions, the participants were instructed to push “as hard as possible” over the entire range of motion. Two min of recovery were maintained between isokinetic velocities, and 30 s were interposed between consecutive contractions within the same angular velocity. Since we were interested in obtaining the highest fascicle shortening velocity during each isokinetic trial, the participants were asked to stay fully relaxed before contraction (i.e., without preload). This procedure agrees with Hauraix et al. [17, 18], which found higher fascicle shortening velocity without preloading.

**FIGURE 1:**
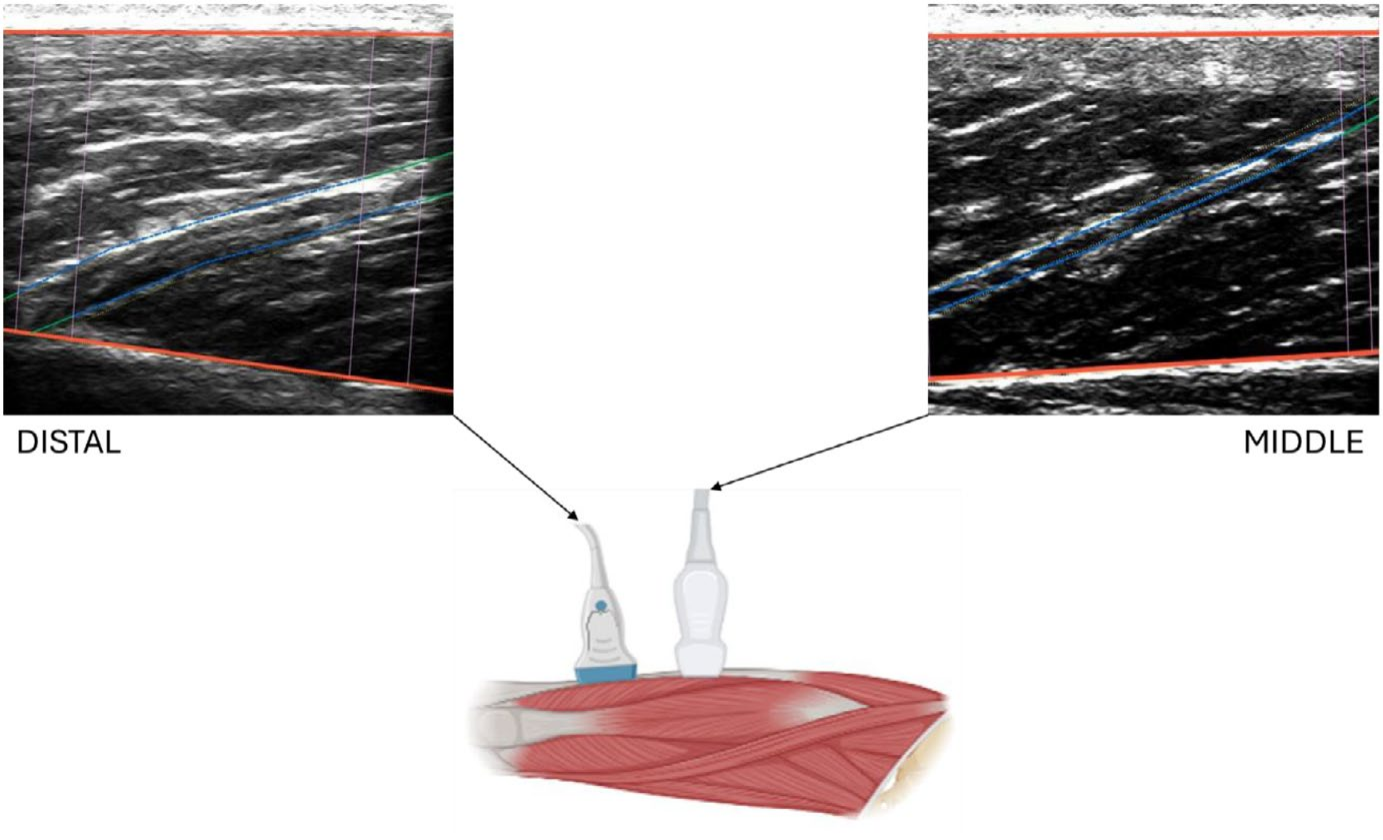
Two ultrasonographic probes were positioned on the vastus lateralis muscle in different regions. Ultrasound images represent the distal and the middle region at rest. The red lines represent the superficial and deep aponeurosis; the blue lines represent the visible portion of the fascicles; the green lines represent the extrapolated fit.

During the resting measurements and during all contractions, fascicle length changes were recorded using two ultrasound apparatuses (MicrUs and ArtUs, Telemed) with a sampling frequency of 45 and 35 Hz (and a field of view of 6 and 4.5 cm), respectively. The longer probe was placed on the distal part of the VL (approximately at 85% of the femur length, from the great trochanter), while the shorter probe was placed in the middle of the muscle belly (at 50% of the femur length) (see Figure 1). The probes were aligned on the muscle belly and adjusted to have a clear image of the perimysial connective intramuscular tissue, indicative of the muscle fascicle structure. The probes were fixed to the skin using a custom‐made plastic involucrum and never removed during the experimental session.

Torque, angular velocity, and angular position were recorded simultaneously using an analog‐digital converter (PowerLab version 8/35); the ultrasound trigger (square wave pulses) was utilized to synchronize the apparatuses.

### 2.4 Data Analysis

The total torque generated by the knee extensors was corrected for the gravitational torque effects (determined during a passive joint rotation driven by the dynamometer) [19]. For the isokinetic contractions, the maximum value of torque reached during the phase at constant angular velocity was determined (see Figure S1), averaged among trials at the same angular velocity, and then used in further analysis.

A validated custom‐made semiautomatic software [20] was used for the ultrasound measurements to quantify fascicle length, muscle thickness, and pennation angle frame by frame (for both regions), considering fascicle curvature. The interested reader is referred to that paper for a detailed description of all the data collection and analysis steps.

Briefly, the software utilized a manual‐linear extrapolation method, where the visible part of the fascicle was divided into four different segments to consider the curvature. The rest of the fascicle, not included in the field of view, was linearly extrapolated, as Franchi et al. [5] suggested. Hence, the fascicle length was calculated as the sum of the four segments plus the linear extrapolation. Muscle thickness was defined as the average distance between superficial and deep aponeurosis, taken at each fascicle's most proximal and distal visible point. Pennation angle was finally obtained as the average of the angle between the deep aponeurosis and a point placed at 20% of the length of the fascicle and the angle between the superficial aponeurosis and a point placed at 80% of the length of the fascicle (to avoid the curvature that is sometimes visible in the distal fascicles). Belly length was measured along the deep and superficial aponeuroses using the intersection points of the fascicle with both aponeuroses. After tracking, every frame was visually examined to check the operator's accuracy; a specific frame was reanalyzed whenever the parameters were deemed inaccurate. Two fascicles were tracked for each condition.

Muscle geometry at rest was evaluated, and three frames were tracked approximately one second before contraction. During contraction, the ultrasound videos were only analyzed during the phase at constant angular velocity, excluding the video frames related to the acceleration and deceleration phases. Typical tracings of muscle thickness (MT), fascicle length (FL), and pennation angle (PA) (during an isokinetic contraction at 30° s^−1^, 150° s^−1^, and 270° s^−1^) are reported in Figure S2.

The fascicle and belly velocities during the phase at constant angular velocity were calculated as the first derivative of the fascicle and belly length changes, respectively. As calculated, muscle‐belly length changes do not represent the entire length change of the VL muscle belly but the longitudinal length change of the VL fascicle length to the plane of the muscle‐tendon unit (MTU) [21]. Both fascicle and muscle‐belly velocities were filtered using a low‐pass (5 Hz) 2nd‐order Butterworth filter (see Figure S3). In the phase at constant angular velocity, the belly gearing (Gb) was calculated as the ratio between muscle‐belly and fascicle velocity (Vm/Vf).

Finally, we calculated the muscle architectural changes as the delta between the last frame of the phase at constant angular velocity and the resting value.

### 2.5 Statistics

Data normality and homoscedasticity were assessed using Shapiro–Wilk and Levene tests, respectively. To examine regional differences in muscle architecture at rest, we used paired sample *t*‐tests. A two‐way repeated measures ANOVA was used to identify significant differences between regions (middle vs. distal) as a function of angular velocity (30° s^−1^, 75° s^−1^, 150° s^−1^, 210° s^−1^ and 270° s^−1^). The alpha level was set at 0.05, and the statistical analyses were performed using SPSS (v.23, IBM Corporation, USA).

## 3 Results

The maximum knee extensors' torque values reached during the phase at constant angular velocity were: 212.6 ± 31.6, 168.5 ± 34.0, 133.1 ± 38.5, 105.7 ± 31.5, 95.5 ± 26.2 N m at 30° s^−1^, 75° s^−1^, 150° s^−1^, 210° s^−1^, and 270° s^−1^, respectively. The knee extensors' torque was affected by knee angular velocity (main effect of velocity: *p* < 0.001): the higher the angular velocity, the lower the torque.

**FIGURE 2:**
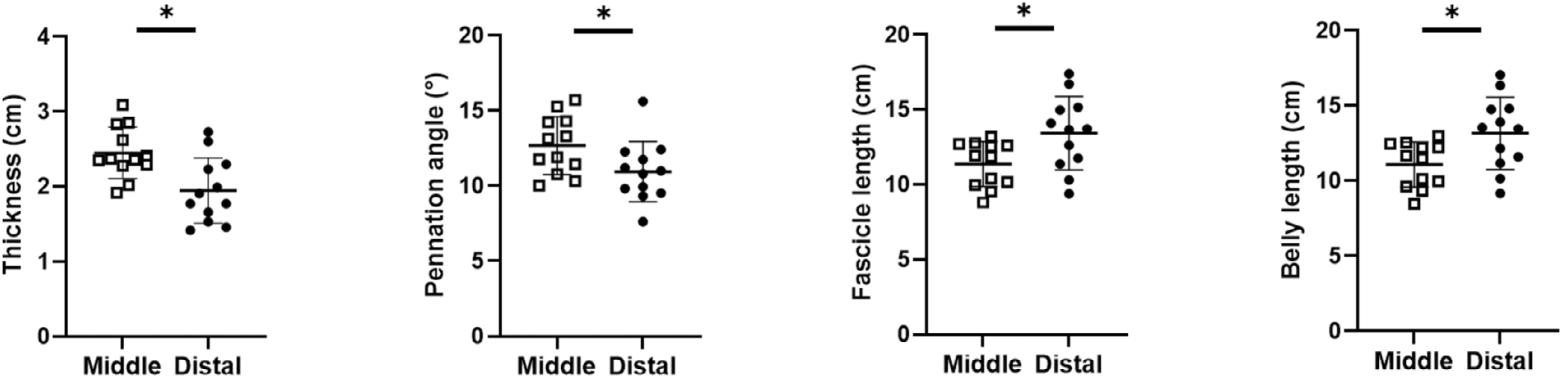
Muscle architecture at rest in the middle (open squares) and distal (dots) region of VL. Significant differences (*) were observed in thickness, pennation angle, fascicle length, and belly length. Values are mean ± SD; individual values are reported as well.

At rest, the distal region showed smaller muscle thickness (*p* = 0.0003), higher pennation angle (*p* = 0.026), and longer fascicle and muscle‐belly lengths (*p* = 0.025 and *p* = 0.024, respectively) compared to the middle one (see Figure 2).

**FIGURE 3:**
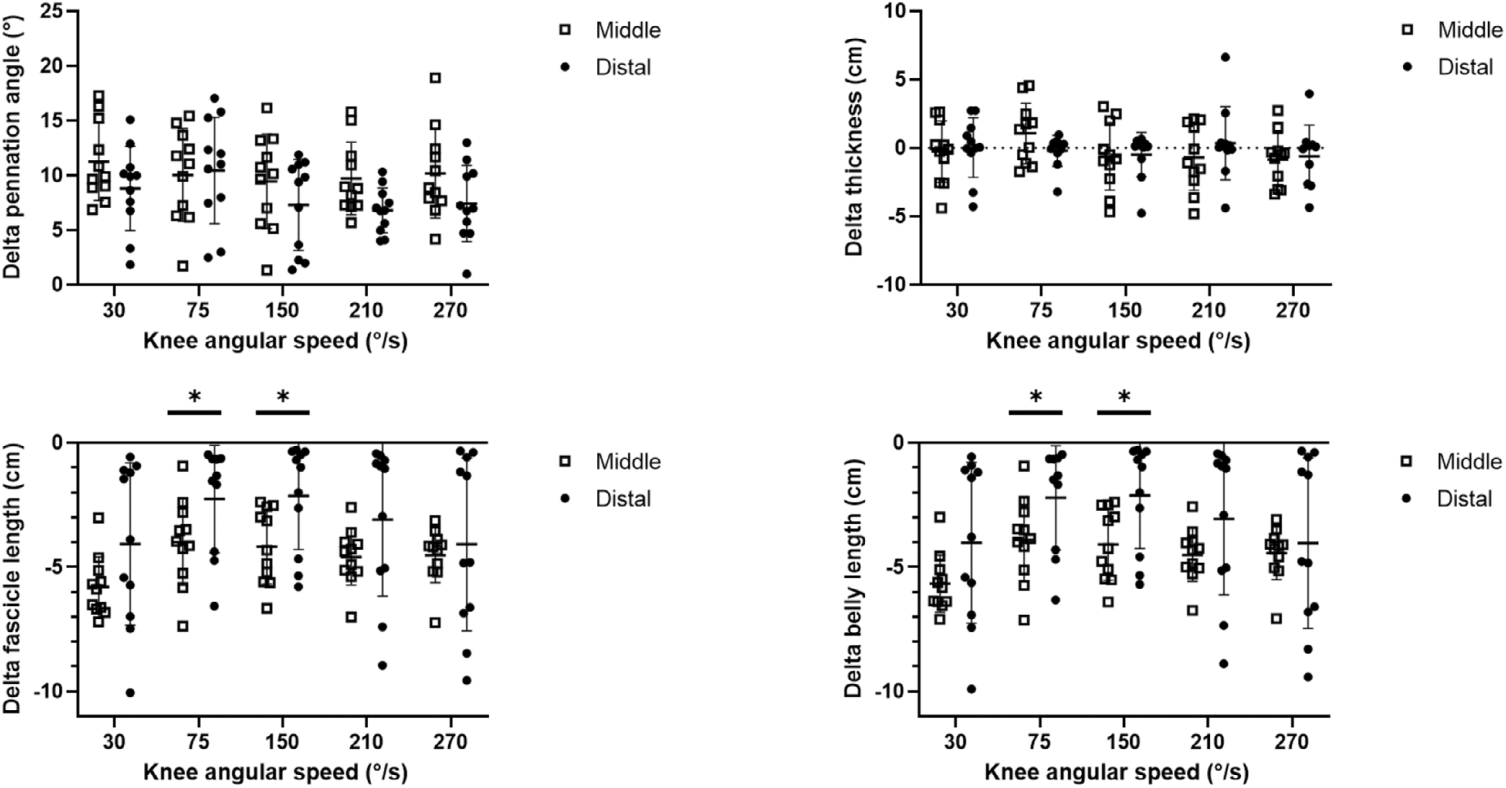
Architectural changes (delta between the last frame of the phase at constant angular velocity and the resting value) in the middle (open squares) and distal (dots) region of VL. Significant changes (*) were observed in pennation angle and muscle thickness but not in fascicle and belly length. Values are mean ± SD; individual values are reported as well.

Muscle architectural changes (delta between the last frame of the phase at constant angular velocity and the resting value) as a function of knee angular velocity are reported in Figure 3. No changes in muscle thickness and pennation angle were observed between regions. Differences between regions were, instead, observed in fascicle and belly length (main effect of the region: *F* = 5.15, *p* = 0.04 and *F* = 4.80, *p* = 0.049, for fascicle and belly length, respectively) at 75° s^−1^ and 150° s^−1^.

**FIGURE 4:**
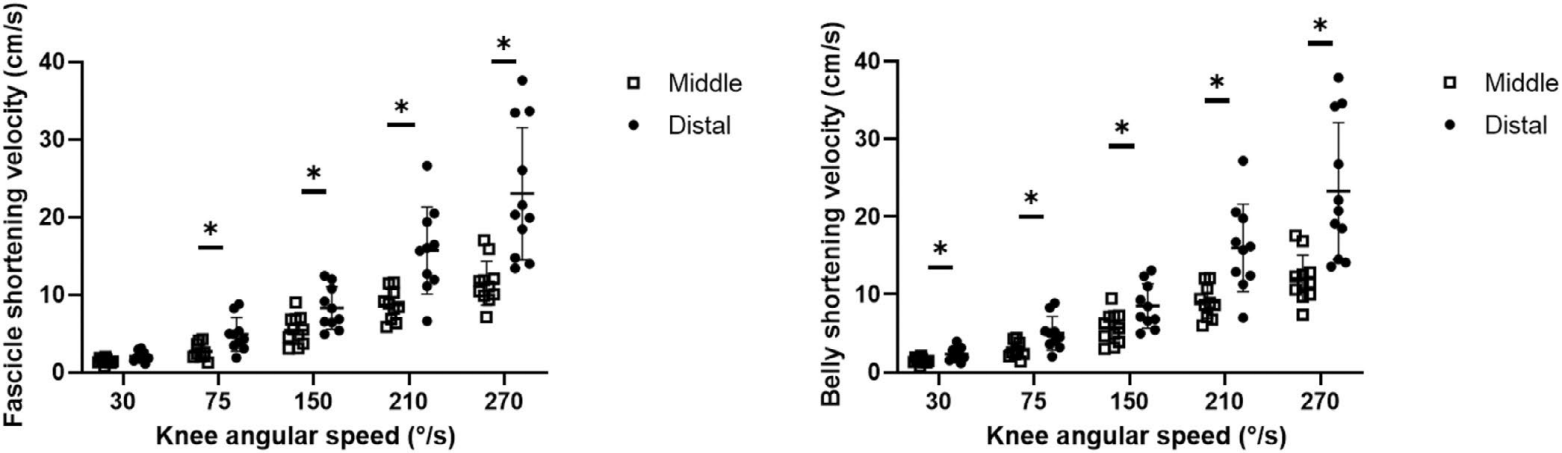
Fascicle and belly shortening velocity during the isokinetic contractions (in the phase at constant angular velocity) in VL's middle (open squares) and distal (dots) region. Values are mean ± SD; individual values are reported as well. *significant differences between regions.

Fascicle and muscle belly velocities increased as a function of knee angular velocity (main effect of velocity: *F* = 41.48, *p* < 0.001 and *F* = 57.21, *p* < 0.001, for Vf and Vm, respectively) in both regions (see Figure 4). Fascicle and muscle belly velocities were higher in the distal region compared to the middle one (main effect of region: *F* = 14.95; *p* = 0.008 and *F* = 22.91, *p* = 0.001, for Vf and Vm, respectively). The post hoc test results are reported in Figure 4.

**FIGURE 5:**
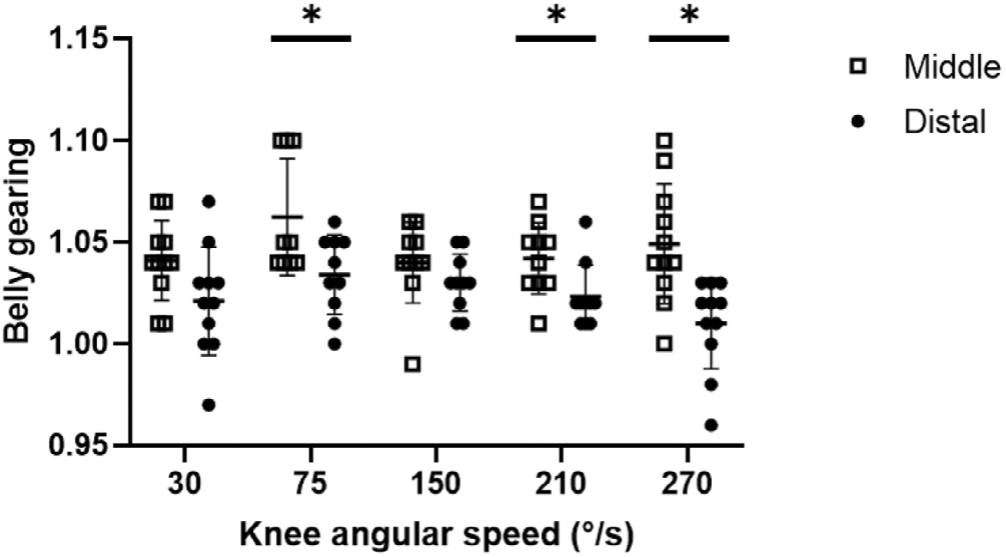
Belly gearing during the isokinetic contractions (in the phase at constant angular velocity) in VL's middle (open squares) and distal (dots) regions. Values are mean ± SD; individual values are reported as well. *significant differences between regions.

Significant differences in belly gearing were observed between regions (main effect: *F* = 8.54, *p* = 0.019) but not as a function of knee angular velocity (*F* = 1.74, *p* = 0.13) (see Figure 5). Post hoc tests showed significant differences in Gb between regions at 75° s^−1^, 210° s^−1^, and 270° s^−1^.

## 4 Discussion

This study investigated how regional differences in muscle architecture influence fascicle and muscle‐belly behaviour during in vivo isokinetic contractions. Our data confirm our first hypothesis that fascicles' velocities differ along the muscle (are larger in the distal than in the middle region). In addition, we observed that these differences are larger at the faster angular velocities (e.g., at the lower torque values).

Our second hypothesis was also confirmed: the higher pennation angles at rest observed in the middle region result in a higher belly gearing compared to the distal region.

Regional heterogeneity in fascicle behavior during contraction is well reported in animal studies: The fascicles in different regions operate in different portions of the force–length and force–velocity relationships [8, 9]. However, this is the first study that demonstrated this mechanism in humans (Figure 5).

The regional differences in fascicle behavior reported in the current study could be attributed to several mechanisms. For example, in accordance with previous literature, we observed longer fascicles at rest in the distal region and higher resting pennation angles in the middle region [5, 6]. These differences in resting muscle architecture are expected to translate into differences in fascicle behavior. Indeed, Lieber and Friden [4] observed that longer fascicles can reach higher shortening velocities than shorter ones. Moreover, it was recently reported by Van Hooren et al. [15] that higher values of pennation angle at rest are correlated to higher fascicle rotation and higher belly gearing during contraction. Our data support this observation, showing that the region with the highest resting pennation angle had the highest belly gearing. Therefore, our findings confirm the observations of Azizi and Deslauriers [9], who suggested that regional variations in muscle and fascicle behavior could be explained by differences in muscle architecture along the muscle at rest.

Another potential mechanism that could explain the observed regional differences could be related to variations in muscle activation along the muscle. In the present study, we did not evaluate muscle activation for technical reasons (e.g., insufficient space for the EMG electrodes since we used two ultrasound probes). However, differences in activation patterns along the same muscle were reported in the literature. For example, Wakeling [22] used surface electromyography (EMG) to evaluate the regional differences in EMG activity of the plantar flexor muscles during cycling at different resistances and cadences. The authors showed variations in the magnitude and timing of the EMG signal in both the proximo‐distal and mediolateral directions, reporting that the regional difference in EMG activity is related to the mechanical request. This specific mechanism should be investigated in the future, possibly in combination with ultrasound data.

A third mechanism that could explain the difference in regional fascicle behavior is the heterogeneity in muscle histological properties. For example, it is common to observe a decreasing gradient of slow‐oxidative fibers from the deep to the most superficial portion or proximal to the distal region of a muscle [23]. According to the size principle of recruitment, slow‐oxidative fibers will be recruited first, and the force will be transmitted from the active muscle fibers to the passive muscle fibers. However, this is not the case for VL, for which no significant differences in fiber type distribution are observed along its longitudinal length [24].

A further mechanism could be related to differences in aponeurosis stiffness along the muscle observed in different experimental animal models. For example, Wheatley et al. [25] showed regional differences in the microstructural characteristics of the porcine triceps brachii aponeurosis when the analysis was conducted close to the tendon or in the middle of the muscle belly. These microstructural differences were also associated with differences in aponeurosis modulus (stiffness) between regions, which could affect fascicle dynamics. To our knowledge, no data about differences in aponeurosis stiffness along the human VL are reported in the literature. We observed comparable muscle belly length changes between regions at all the investigated contraction velocities, and this suggests that no significant differences in aponeurosis stiffness could be expected between the middle and distal regions during maximum isokinetic contractions. As previously indicated by Azizi and Deslauriers [9] in animal models, the geometrical differences between regions are the most critical mechanisms explaining regional heterogeneity in fascicle behavior. Our data indicate that this is the case also for human muscles in vivo, providing important implications for musculoskeletal models.

Significant differences between regions were also observed in the uncoupling behavior between the muscle belly and its fascicles (e.g., in belly gearing). Gb is an essential functional parameter that could affect mechanical (e.g., explosive force production, mechanical power, and muscle force) [13, 15, 26] and physiological behavior (e.g., the energy cost of locomotion and the metabolic power of a given contraction) [14, 27, 28]. For example, for a given contraction, a decrease in belly gearing was associated with an increase in the metabolic power needed to produce a given mechanical power, affecting contraction efficiency [14]. Belly gearing is the highest during low‐force and high‐velocity contractions and reduced during high‐force–low‐velocity contractions. Thus, according to the F–V relationships, an increase in belly gearing can be expected as a function of isokinetic velocity; however, in the current study, this was not observed. Although most of the literature reports a significant effect of muscle force on belly gearing (i.e., the higher the force, the lower the belly gearing) [29], some studies show a different behavior [30]. For example, in agreement with our data, Randhawa and Wakeling [31] observed no significant changes in the gastrocnemius lateralis belly gearing as a function of isokinetic velocity. In contrast, Kelp et al. [32] observed an increase (rather than a decrease) in the gastrocnemius medialis belly gearing as a function of muscle contraction intensity (isometric contractions). The reason behind these differences is not immediately apparent and needs to be clarified.

However, it is well known that a muscle operates with a low belly gearing when the thickness of the muscle decreases, a moderate belly gearing when the thickness of the muscle is constant, and a high belly gearing when the thickness of the muscle increases during contraction [29]. As reported in Figure 3, the changes in muscle thickness (delta between the last frame of the phase at constant angular velocity and the resting value) are negligible and do not change as a function of contraction velocity. Therefore, low or moderate values of belly gearing could be expected, with no significant changes as a function of angular velocity, as indeed found in this study.

The significant differences in belly gearing between regions suggest the uncoupling behavior between the muscle belly and its fascicle could be region‐dependent. A functional benefit of muscle architectural heterogeneity could be related to the effects on the force–length relationship. The differences in fascicle length observed in this study between regions could imply that different fascicles will reach their optimal length for force generation at different overall muscle lengths, potentially increasing the plateau of the muscle force‐length curve [2]. This would lead to the entire muscle operating more effectively over various lengths; alternatively, muscles that are architecturally more homogeneous would be more “specialized” and could only produce (high) force over a narrow range of lengths.

Finally, muscle heterogeneity could be related to differences in the force–velocity potential (i.e., the fraction of the maximum force that a muscle could attain at a given shortening velocity). Indeed, since a high belly gearing is associated with a high force–velocity potential [14], we could expect a lower force potential for the muscle fascicle located in the distal region. This mechanism has physiological relevance since a decreased force–velocity potential was associated with increased metabolic energy needed to sustain a contraction and a reduced muscle contraction efficiency [14, 33]. Indeed, regional differences in oxygenation status, consistent with regional differences in muscle architecture, were observed in GM using functional near‐infrared spectroscopy (NIRS) [34]: the energy cost of contraction being higher (and oxygen saturation lower) in the distal part of the muscle.

## 5 Limitations and Further Considerations

Muscle geometry and fascicle dynamics were assessed only in the VL. Although the VL is regarded as a primary knee extensor due to its sizeable cross‐sectional area, the other quadricep compartments contribute to the knee extensor moment [35].

2D ultrasonography represents a simplified approach compared to the complex 3D architecture of the vastus lateralis [36] (and 3D behavior of muscles during contraction in general) since gearing can occur both in the sagittal and the coronal plane [37].

The torque generated by the knee extensors also depends on the contribution of the antagonist muscles. This contribution was not accounted for in this study because: (i) we were unable to collect EMG data from the knee flexors for lack of space along the muscle (we used two ultrasound probes) and (ii) reliable EMG data from the antagonist muscles could not be collected as well since (with our setup) the participants would sit on the electrodes, affecting signal quality. However, in a previous study, with a setup and procedures like those adopted, we estimated that the contribution of the antagonist's muscles in healthy, active participants was < 5% [38].

Ultrasound data were collected with two probes of different sizes. Although the more extended probe was placed on the distal region to follow better the curvilinear path of the fascicles, the linear extrapolation is not free from potential errors [20]. In future studies, similar probes should be used in the two regions.

## 6 Practical Perspectives

Our data confirm previous observations regarding regional anatomical differences in muscle architecture at rest in humans; we further observed that these differences relate to differences in fascicle behavior during in vivo contractions. Our results could help better understand the effects of eccentric training and stretching protocols that induce different adaptations (in thickness, pennation angle and fascicle length) in a muscle's middle or distal region [39]. In addition, these data could help develop new musculoskeletal models that can better predict the muscle's mechanical output.

## Author Contributions

M.T. and A.M. participated in the study's design, contributing to data collection, analysis, and interpretation. B.B. participated in and contributed to data collection, analysis, and interpretation. P.Z. participated in the study's design, material acquisition, and data interpretation, and supervised the project. All authors contributed to the manuscript writing. All authors have read and approved the final version of the manuscript.

## Conflicts of Interest

The authors declare no conflicts of interest.

## Supporting information


Figure S1.–S3.

## Data Availability

The data supporting this study's findings are available from the corresponding author upon reasonable request.
