# Supplementary material for: Regional Heterogeneity in Vastus Lateralis Architecture Influences Fascicle Behavior During In Vivo Contractions
Source: Scand J Med Sci Sports. 2025 Jul 9;35(7):e70103. doi: 10.1111/sms.70103 (PMC12239164; doi:10.1111/sms.70103)

Supplementary material

Michele Trinchi, Baptiste Bizet, Paola Zamparo^*^, Andrea Monte

**Regional heterogeneity in vastus lateralis architecture influences fascicle behaviour but not force-velocity potential during in-vivo contractions**

**Figure S1**: Angular velocity (green line) and knee joint torque (orange line) as a function of contraction duration at three angular speeds (top panel: 30°/s; central panel: 150°/s; bottom panel: 270°/s). Vertical dashed (red) lines refer to the start and the end of the isokinetic phase. The (peak) torque values were calculated within the iso-velocity phase; the acceleration and deceleration phases were thus excluded from data analysis.


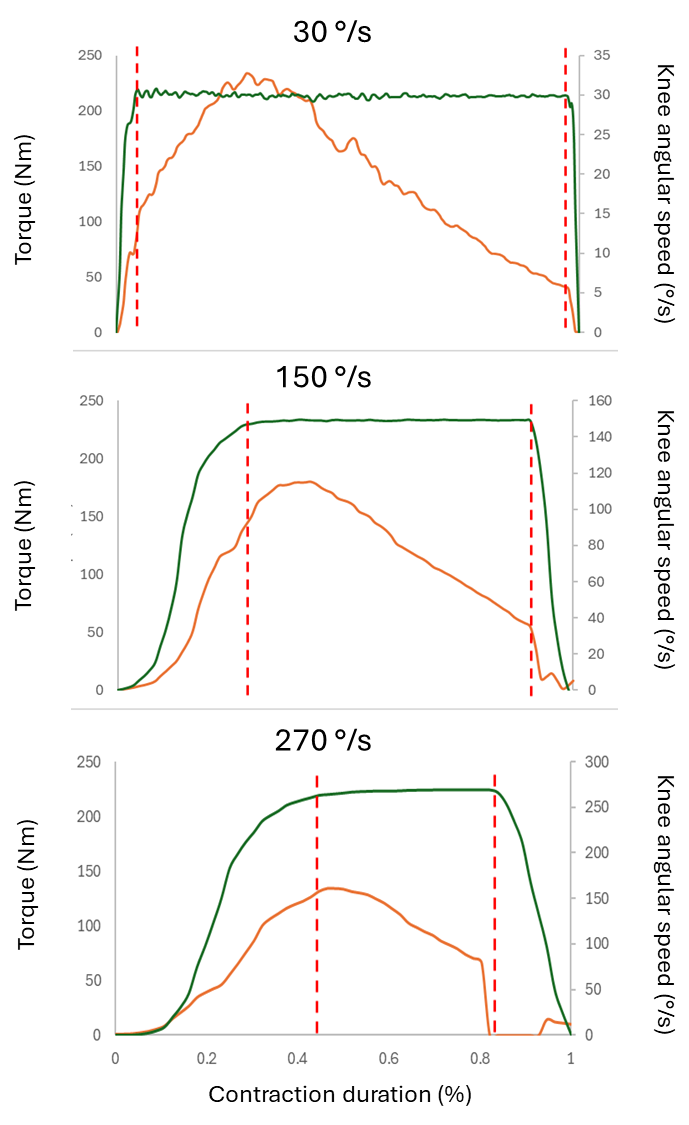


**Figure S2:** Pennation angle and fascicle length changes in the middle (continuous line) and distal (dotted line) regions of VL during the isokinetic phase at three angular speeds (left panel: 30°/s; central panel: 150°/s; right panel: 270°/s).


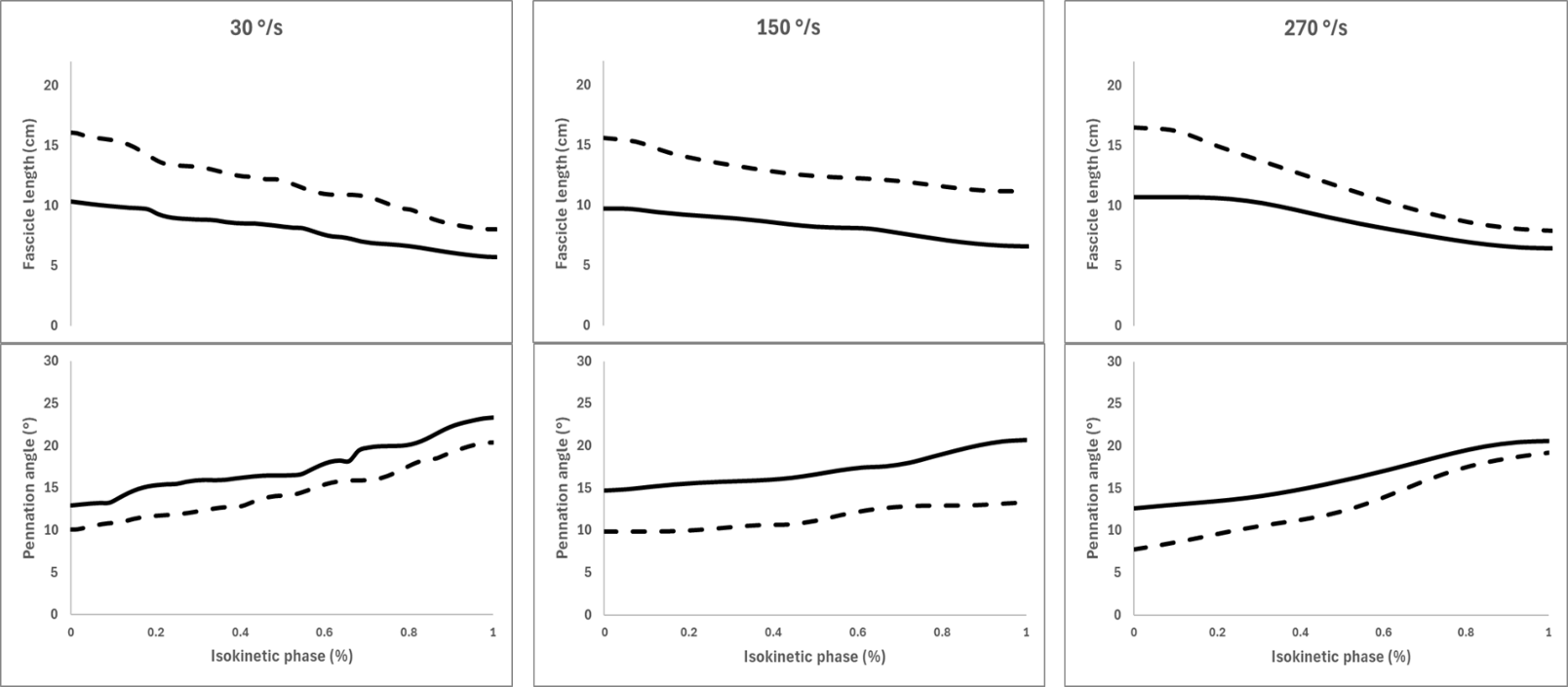


**Figure S3.** Time course of torque, knee angular velocity, fascicle length and fascicle velocity of vastus lateralis during a maximal isokinetic contraction at 75◦/s of angular velocity. Vertical black bars indicate the steady state of isokinetic velocity where the US data were analyzed (fascicle length and fascicle velocity data are indeed reported just in this interval).


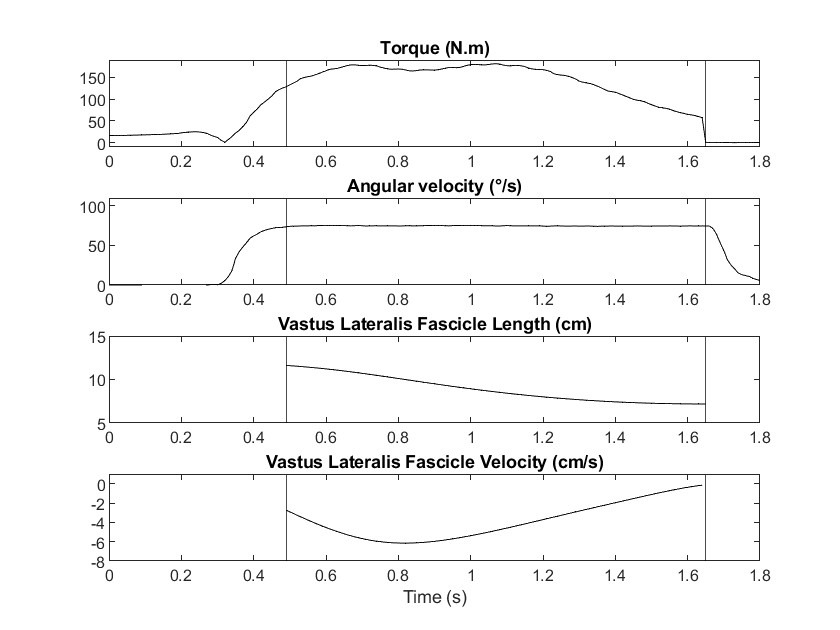


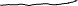

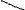

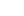

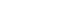

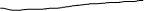

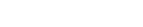

Supplement: Supplementary file 1 — Figure S1.–S3. [file SMS-35-e70103-s001.docx]
